# Muno-IgY Supplementation Improves Respiratory Health, Immune Response, and Exercise-Induced Physiological Stress in Healthy Adults: A Randomized Controlled Pilot Study

**DOI:** 10.3390/nu18030524

**Published:** 2026-02-04

**Authors:** Shahna Fathima, Paul E. Kilgore, Tina Sarkar, Navneet Sharma, Huan H. Nguyen

**Affiliations:** 1 IGY Immune Technologies & Life Sciences Inc., 4 East Lake Ave NE, Unit 1A, Airdrie, AB T4A2G8, Canada; tina.p.sarkar@gmail.com (T.S.); nsharma@igylifesciences.com (N.S.); huan.nguyen@igylifesciences.com (H.H.N.); 2 Clinical Research Center, CS Mott Center for Human Growth and Development, Wayne State University, Detroit, MI 48201, USA; paul.kilgore@wayne.edu; 3 Center for Emerging and Infectious Diseases, Wayne State University, Detroit, MI 48201, USA; 4 Department of Dermatology, University Medical Center Mainz, 55131 Mainz, Germany

**Keywords:** immunoglobulin Y, gut microbiome, nutritional immunology, nutraceuticals, quality of life, immune response, gut health, gut–lung axis, mucosal immunity, inflammation

## Abstract

Background/Objectives: Upper respiratory tract infections (URTIs) and exercise-induced immune perturbations are common in adults and may adversely affect quality of life, productivity, and physical performance. Immunoglobulin Y (IgY), a food-derived antibody with broad antimicrobial activity, has demonstrated immunomodulatory potential in preclinical and limited clinical studies. This study evaluated the effects of a multi-pathogen-specific IgY supplement (Muno-IgY) on respiratory health, immune and inflammatory markers, exercise-induced physiological stress, and gut microbiome composition in healthy adults. Methods: In this 12-week, double-blind, placebo-controlled trial, 28 healthy adults with a history of URTI were randomly allocated to receive Muno-IgY or placebo and URTI incidence, duration, and severity were recorded daily. Serum immune and inflammatory biomarkers were assessed longitudinally and in response to a standardized exercise challenge. Gut microbiome composition was analyzed using shotgun metagenomic sequencing at baseline and week 12. Safety and tolerability were assessed throughout the study. Results: URTI incidence was lower in the Muno-IgY group compared with placebo (14.3% vs. 35.7%), with shorter average duration and fewer missed workdays, though differences were not statistically significant (*p* > 0.05). Following an acute exercise challenge, Muno-IgY supplementation resulted in a significant increase in serum IgA at 24 h post-exercise (*p* = 0.022) and a significantly greater reduction in lactate dehydrogenase at 1 h post-exercise compared with placebo (*p* < 0.0001). Exploratory gut microbiome analyses suggested favorable directional shifts, though these changes were not statistically tested. Conclusions: In this exploratory pilot study, Muno-IgY supplementation was safe and associated with significant improvements in selected markers of exercise-induced immune response and muscle damage. Numerical trends in URTI incidence and gut microbiome composition were observed but were not statistically significant. These findings are hypothesis-generating and support further evaluation of Muno-IgY in larger, adequately powered clinical trials.

## 1. Introduction

Upper respiratory tract infections (URTIs), including laryngitis, pharyngitis, nasopharyngitis, and rhinitis, are among the most common conditions presenting in primary care. Patients are typically presented with symptoms including sore throat, nasal obstruction, cough, and headache. Though the condition is usually self-limiting, the symptoms significantly impair the quality of life and productivity of the patients [1] by delaying mental processing, reducing attentiveness, hedonic tone, and semantic memory reaction time, all of which significantly impact everyday tasks and professional duties [2], ultimately resulting in absence from school and work.

Respiratory illness also poses a significant burden in athletic populations. During the Winter Olympic Games 2010, a study reported that respiratory illnesses accounted for 62.8% of all illnesses in athletes. Although the frequency of URTIs in athletes is comparable to the general population, the episodes are frequently associated with increased training load and the time around competitions, significantly affecting performance, muscular coordination, alertness and information processing [3] and are associated with suppressed mucosal and cellular immunity [4]. Conventional treatments largely address the symptoms of URTI rather than prevention. Hence, there is growing interest in safe, natural interventions that can support immune resilience.

In recent decades, research on the human microbiome has expanded substantially, providing strong evidence that host-associated microbes are critical for the development and maturation of the immune system and play a central role in both health and disease. The intestinal microbiota can influence immune homeostasis at distant mucosal sites, including the respiratory tract, through the integrated networks of the common mucosal immune system [5]. Furthermore, gut microorganisms contribute to the maintenance and differentiation of T-lymphocyte subsets residing in distal mucosal tissues, underscoring the importance of gut–immune crosstalk in shaping systemic and respiratory immunity [5,6].

Preclinical investigations further support the central role of the gut microbiota in respiratory health [7]. In murine models of pneumococcal pneumonia, depletion of the gut microbiota resulted in greater bacterial dissemination, heightened inflammation, increased organ damage, and higher mortality, which resolved through fecal microbiota transplantation. Whole-genome profiling of alveolar macrophages from microbiota-depleted mice revealed marked upregulation of metabolic pathways associated with impaired cellular responsiveness to microbial stimuli such as lipopolysaccharide and lipoteichoic acid, and reduced ability to phagocytose *Streptococcus pneumoniae* [7]. Collectively, these findings highlight the profound influence of the gut microbiota on pulmonary immune function and reinforce the importance of interventions that support gut–lung immune crosstalk. As a result, gut health interventions, particularly supplements modulating gut microbiome, have been widely explored for their potential to enhance mucosal immunity and lower URTI risk [5,8,9,10].

Immunoglobulin Y (IgY), the functional equivalent of mammalian IgG, is present in the serum of birds, reptiles, and amphibians, and transferred to the embryo through the egg yolk. IgY exhibits broad antibacterial and antiviral binding capabilities and does not activate mammalian Fc receptors or the complement system, enabling immunomodulation without triggering inflammatory cascades. Also, IgY does not induce specific resistance because it is directed against multiple antigenic targets [11]. The proposed mode of action of IgY is mediated through neutralization [12], opsonization [13,14], adherence blockade [13,15], and agglutination [11]. Previous studies have demonstrated the prophylactic and therapeutic effect of IgY in vitro and in vivo in murine models of respiratory infection, and the protective effects observed were comparable to those of the neuraminidase inhibitor oseltamivir [16,17].

However, most previous studies have focused on the effect of IgY derived from hens immunized against a single pathogen and were primarily evaluated using animal models. To address this gap, the present randomized double-blinded pilot study primarily aimed to evaluate the effects of a multi-pathogen-specific IgY product, Muno-IgY, on URTI incidence, duration, and severity. Secondary objectives included assessment of inflammatory and immune biomarkers, and exercise-induced physiological stress, including markers of muscle damage and inflammation. Exploratory outcomes encompassed gut microbiome composition, participant-reported quality of life, gastrointestinal tolerance, and overall safety. By distinguishing primary, secondary, and exploratory endpoints, this study provides a structured, hypothesis-generating evaluation of Muno-IgY’s potential to support immune resilience, modulate the gut microbiome, and facilitate recovery from physiological stress.

## 2. Materials and Methods

### 2.1. Study Design, Participants and Data Collection

This 12-week study was conducted as a two-arm, double-blind, placebo-controlled trial with 1:1 computer-generated randomization at Apex Trials (formerly Nutrasource Pharmaceutical and Nutraceutical Services, Inc.) in Guelph, Ontario, Canada, between December 2022 and July 2023. Healthy adults who had self-reported at least one URTI in the previous six months were eligible for enrollment. A total of 28 participants aged 35–65 years who were employed or attending school for at least 20 h per week were randomized to receive either Muno-IgY^®^ (IGY Immune Technologies and Life Sciences Inc., Thunder Bay, ON, Canada) or placebo. Participants in the Muno-IgY group consumed two capsules daily, providing 100 mg of egg yolk protein (15 mg IgY per capsule), while those in the placebo group received two identically appearing capsules containing rice flour. The dose of Muno-IgY was chosen to evaluate the effects as well as product safety and tolerability. The study products were instructed to be consumed in the morning with or without food.

The Randomization Scheme was computer-generated by Nutrasource using SAS 9.4 PROC PLAN on 22NOV22:15:24:17, and study products were dispensed according to the randomization scheme. Both participants and study personnel, including the investigator, study team, sponsor representatives, data management staff, and statisticians, remained blinded to treatment allocation. Only designated unblinded personnel at the CRO, responsible for labelling, accountability, and reconciliation of study products, had access to treatment assignments. Unblinding was permitted only in emergencies or situations requiring knowledge of the study product, with all instances reported to the investigator, CRO, and sponsor. Planned unblinding occurred after database lock and completion of the blinded statistical analysis.

**Figure 1:**
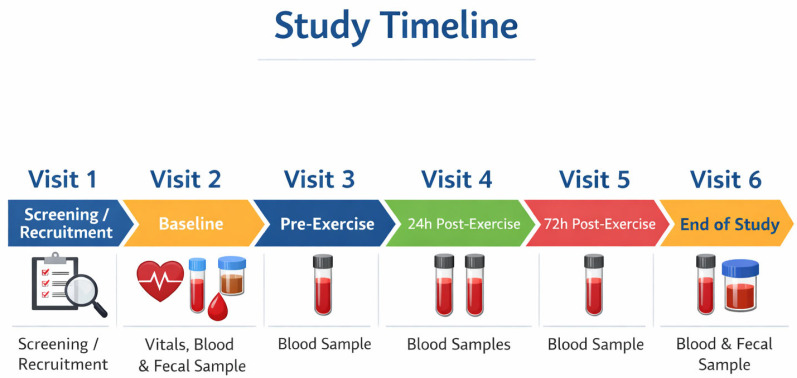
Timeline of study visits and sample collection over 12 weeks.

This study involved six clinic visits, including a screening visit, a baseline visit, three assessments during Week 4, and a final end-of-study visit at Week 12. Figure 1 illustrates the schedule of study visits, including the timing of baseline and post-exercise sample collections. Throughout the intervention period, participants were instructed to record the incidence, duration, and severity of any URTI symptoms using daily logs. A summary of demographic data, body measurements, pregnancy status, and vital signs, including diastolic and systolic blood pressure, heart rate, respiratory rate and body measurements, was obtained at screening. Participant withdrawals were not replaced.

Two questionnaires were used in this study to screen participants. The Physical Activity Readiness Questionnaire (PAR-Q) has been previously validated in clinical trials to assess whether participants were suitable to perform physical activity based on any existing medical conditions [18]. In addition, the International Physical Activity Questionnaire (IPAQ) was used to assess the current physical activity of participants [19].

The trial received ethical approval from the Sterling Institutional Review Board (Sterling IRB—Your Dedicated Institutional Review Board (https://www.sterlingirb.com/, accessed on 24 January 2026), Atlanta, GA; Approval Reference #10578, Approval date 12/12/2022) and authorization from Health Canada’s Natural and Non-Prescription Products Directorate (ISRCTN Registry #51932144). All participants provided written informed consent prior to enrollment, between December 2022 and July 2023.

### 2.2. Safety Assessments on Muno-IgY Supplementation

#### 2.2.1. Height, Weight, and Body Mass Index (BMI)

Height was measured at screening (Visit 1) in centimeters to one decimal place using a stadiometer, with participants barefoot and standing upright. Weight was measured at Visits 1, 2, 3, and 6 in light clothing using a beam scale, with participants wearing light clothing, and recorded in kilograms to one decimal place. Body mass index (BMI) was calculated as weight (kg) divided by the square of height (m) and recorded to one decimal place (kg/m^2^). All measurements were rounded to the nearest tenth following standard conventions.

#### 2.2.2. Vital Signs

Vital signs, including blood pressure, heart rate, and respiratory rate, were measured in a seated position at Visits 1, 2, 3 (pre-exercise) and 6. All measurements were recorded in source documents and case report forms (CRFs).

#### 2.2.3. Laboratory Assessments

Blood samples were collected for safety laboratory assessments at multiple time points. Non-fasted samples were obtained at Visit 1, and fasted samples were collected at Visits 2, 3 (pre-exercise), and Visit 6. Hematology parameters included hemoglobin, hematocrit, white blood cell count with differential (neutrophils, eosinophils, basophils, lymphocytes, monocytes), red blood cell count, red cell distribution width, red blood cell indices (mean corpuscular volume, mean corpuscular hemoglobin, mean corpuscular hemoglobin concentration), platelet count, and mean platelet volume. Clinical chemistry parameters included sodium, potassium, chloride, urea, creatinine, total protein, albumin, total bilirubin, glucose (serum; fasting except at screening), alanine transaminase, aspartate transaminase, alkaline phosphatase, estimated glomerular filtration rate (eGFR), and globulin.

#### 2.2.4. Adverse Events and Safety Monitoring

Adverse events (AEs) were defined as any untoward medical occurrence in a participant receiving the study product, regardless of causality [20]. Symptoms of URTIs were recorded in participant diaries and could include cough, runny or congested nose, sore or scratchy throat, sneezing, hoarseness, lethargy, body aches, and fever. The investigator evaluated symptoms and may have made a clinical diagnosis of URTI based on these reports.

Only new or worsening medical occurrences after study product administration were recorded as AEs. Investigators and designated study staff documented all AEs, including those spontaneously reported, observed, or elicited through questioning, in the participant’s case report form. Participants were followed at subsequent visits for AE monitoring. Table 1 summarizes the key data elements collected for each AE.

Serious Adverse Events (SAEs) were defined as AEs resulting in death, life-threatening conditions, hospitalization, prolonged hospitalization, significant disability, congenital anomaly, or other medically important events [21]. SAEs were recorded in the same manner as AEs and followed until resolution, stabilization, or explanation.

SAEs were reported promptly to the CRO, sponsor, and regulatory authorities. Health Canada’s Therapeutic Products Directorate (TPD) was notified of all serious or unexpected adverse reactions using the Council for International Organizations of Medical Sciences (CIOMS) form, within 7 days if fatal or life-threatening, and within 15 days if neither fatal nor life-threatening, with follow-up reports as needed.

### 2.3. Evaluating the Effect of Muno-IgY on Immune Responses

#### 2.3.1. Effect of Muno-IgY on Incidence, Severity, and Duration of Upper Respiratory Tract Infections

To evaluate the effects of Muno-IgY on immune function, participants self-reported the number of incidences of URTI, as well as the severity and duration of the episode, using a daily diary throughout the study. The duration of URTI was calculated by dividing the total duration of all URTI in a study group by the number of URTI experienced in that study group. The severity of URTI was calculated by dividing the total number of days of work or school missed due to URTI symptoms by the number of URTIs experienced in that study group. A comparison between the Muno-IgY and the placebo group was conducted to evaluate their effects on the number of incidences. No statistical analysis was performed on the duration and severity of URTIs due to the small number of participants who experienced URTIs.

#### 2.3.2. Effect of Muno-IgY on Serum IgA Concentration

The effect of Muno-IgY on immune response in participants was measured by serum IgA levels from whole blood samples collected under fasting conditions at Visits 2, 3 (pre-exercise), 4 and 6. Following blood collection, the whole blood samples were incubated for 30–120 min at room temperature, centrifuged at 1100–1300× *g* for 10–15 min at 18–25 °C, and shipped to an external laboratory (Dynacare, Ontario, Canada) at 2–8 °C for analysis. Serum IgA levels were determined by immunonephelometry on the Optilite platform (The Binding Site, Birmingham, UK; Item # NK010.OPT) following the manufacturer’s protocol. The value obtained from samples collected at Visit 2 was treated as baseline and used to calculate the change from baseline to Visits 3, 4 and 6. A comparison between the Muno-IgY and placebo groups was performed on the change from baseline to differentiate between their effects on immune response.

#### 2.3.3. Effect of Muno-IgY on Inflammatory Biomarkers

Inflammatory biomarkers such as C-reactive protein (CRP), Lipopolysaccharide Binding Protein (LBP), Cortisol, Tumor Necrosis Factor-α (TNF-α), Interleukin-6 (IL-6), Interleukin-10 (IL-10) and Interleukin-1-β (IL-1-β) were quantified using serum separated from whole blood samples collected under fasting conditions at Visits 2, 3 (pre-exercise), 4, 5 and 6.

Following collection, the whole blood samples collected for LBP, TNF-α, IL-6, IL-10, and IL-1β were incubated for 30–60 min at room temperature, centrifuged at 2000× *g* for 10 min at 2–8 °C, and shipped to an external laboratory at −80 °C for ELISA testing (Dynacare, Ontario, Canada). Similarly, the samples collected for CRP and cortisol were incubated for 30–120 min at room temperature, centrifuged at 1100–1300× *g* for 10–15 min at 18–25 °C, and shipped to an external laboratory at 2–8 °C for analysis (Dynacare, Ontario, Canada).

Serum cytokine concentrations were determined using commercially available ELISA kits (Invitrogen, Vienna, Austria) in accordance with the manufacturers’ instructions, including Human IL-6 ELISA (BMS213-2, BMS213-2TEN), Human IL-10 ELISA (BMS215-2, BMS215-2TEN), Human IL-1β ELISA (BMS224-2, BMS224-2TEN), and Human TNF-α High Sensitivity ELISA (BMS223-2HS), Human Cortisol Competitive ELISA (EIAHCOR), Human LBP ELISA (EH297RB), and Human CRP ELISA (KHA0031). Serum CRP was measured using a particle-enhanced immunoturbidimetric assay on the Roche c701/c501 analyzer (Roche Diagnostics, Indianapolis, IN, Item #7876424190) and fasting serum cortisol was determined by electrochemiluminescence immunoassay (ECLIA) on the Roche e801/e601 analyzer (Roche Diagnostics, Indianapolis, IN, Item #7027150190), following the manufacturer’s instructions.

The data from samples collected at Visit 2 were treated as baseline and used to calculate the change from baseline to Week 4 and Week 12 (Visit 6). A comparison between the Muno-IgY and placebo groups was made on the change from baseline to Weeks 4 and 12 to differentiate between their effects on inflammatory biomarkers.

### 2.4. Effect of Muno-IgY on Post-Exercise Inflammatory, Immunity and Muscle Damage Responses

Inflammatory biomarkers such as CRP, Cortisol, TNF-α, IL-6, IL-10, and IL-1β, markers of muscle damage such as CK and LDH, and IgA concentration were quantified using serum separated from whole-blood samples collected under fasting conditions at visit 3 (pre-exercise, and 1 h ± 5 min and 2 h ± 5 min post-exercise), 4 (24 ± 1 h post-exercise) and 5 (72 ± 1 h post-exercise). The samples were processed as described above and sent to an external laboratory for analysis (Dynacare, Ontario, Canada).

Serum CK and LDH activities were measured enzymatically using a spectrophotometric method on the Roche c701/c501 analyzer (Roche, Item #5168546190 for CK; Item #5169330190 for LD) following the manufacturer’s instructions. Serum cytokine concentrations were determined using commercially available ELISA kits (Invitrogen, Vienna, Austria) in accordance with the manufacturers’ instructions, including IL-6 (BMS213-2, BMS213-2TEN), IL-10 (BMS215-2, BMS215-2TEN), IL-1β (BMS224-2, BMS224-2TEN), TNF-α (BMS223-2HS), and LBP (EH297RB). Serum IgA levels were determined by immunonephelometry on the Optilite platform (The Binding Site, Birmingham, UK; Item # NK010.OPT) following the manufacturer’s protocol. Similarly, serum CRP was measured using a particle-enhanced immunoturbidimetric assay on the Roche c701/c501 analyzer (Roche, Item #7876424190) and fasting serum cortisol was determined by electrochemiluminescence immunoassay (ECLIA) on the Roche e801/e601 analyzer (Roche, Item #7027150190), following the manufacturer’s instructions.

The exercise challenge consisted of a standardized quadriceps fatigue protocol performed on a leg-press machine [22] under certified trainer supervision. Samples collected before exercise at Visit 3 were used as the exercise baseline and were used to calculate the change from the exercise baseline to post-exercise. The magnitude of change from baseline was compared between the Muno-IgY and placebo groups to evaluate differences in inflammatory, immune, and muscle-damage responses following the exercise challenge.

### 2.5. Effect of Muno-IgY Supplementation on Gut Microbiome

Fecal samples were collected from study participants at Week 2 (baseline) and Week 12 (end of study). Samples were processed for shotgun metagenomic analysis by Microbiome Insights (Richmond, BC, Canada). Total genomic DNA was extracted using the NucleoMag DNA Microbiome Kit (4 × 96 format) (Macherey-Nagel, Düren, Germany), optimized for automated extraction on the Thermo Fisher Scientific KingFisher platform, in accordance with the manufacturer’s instructions [23]. DNA quality control was performed prior to library preparation.

Shotgun metagenomic libraries were generated from extracted DNA and sequenced using the Illumina NovaSeq platform with a high-output configuration (2 × 150 bp paired-end reads). Sequencing targeted an average depth of approximately 12 million reads per sample, with at least 90% of samples achieving a minimum of 5 million reads following quality control. Bioinformatic processing, taxonomic profiling, and report generation were conducted by Microbiome Insights (Richmond, BC, Canada) using validated internal pipelines. Analyses were restricted to bacterial taxa; non-bacterial reads, including viral and other non-target organisms, were excluded prior to downstream analyses.

Changes in microbial abundance were evaluated by comparing relative abundance between Week 2 (baseline) and Week 12 (end of study) within each treatment group.

## 3. Statistical Analysis

All statistical analyses were performed using RStudio (R version 4.5.1). Incidence of URTIs was summarized as the number and percentage of participants experiencing an event per treatment group. Between-group differences in incidence were evaluated using Fisher’s exact test, and average URTI incidence per participant was compared using the Wilcoxon rank-sum test. No formal statistical analysis was performed for URTI severity due to the small number of affected participants.

Changes from baseline (pre-intervention or pre-exercise values) for serum markers of immunity, muscle damage, and inflammation were calculated for each parameter. Repeated measures analysis was conducted using linear mixed-effects models with Treatment and Time as fixed effects and Subject as a random effect to account for within-subject correlations. Type III ANOVA with Satterthwaite’s method was used to calculate F-statistics and *p*-values. Post hoc pairwise comparisons at each time point were performed using estimated marginal means with Tukey adjustment for multiple comparisons. Results are presented as mean ± standard error of the mean (SEM). Statistical significance was defined as *p* < 0.05.

For microbiome analysis, mean relative abundances of the taxa were calculated per treatment group. Differential abundance analyses were performed using Kruskal–Wallis tests with false discovery rate (FDR) correction (Benjamini–Hochberg). LEfSe-style criteria were applied to flag taxa with absolute log_2_ fold-change ≥ 2 and −log_10_(*p*) > 1.3 as potentially relevant. Alpha (Shannon, Simpson) and beta diversity (Bray–Curtis) analyses were conducted, and PERMANOVA was used to assess group differences. All microbiome results are reported descriptively, as this study was exploratory and not powered for formal statistical significance at the species level.

## 4. Results

### 4.1. Study Population and Baseline Characteristics

**Figure 2:**
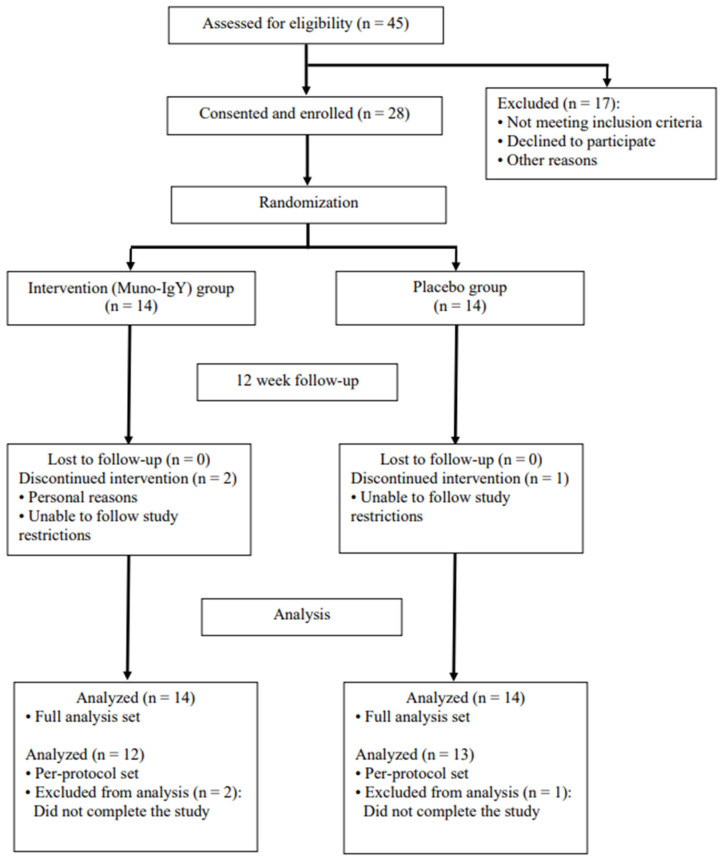
CONSORT flow diagram illustrating participant enrollment, randomization, allocation, follow-up, and analysis in the two-arm, randomized, placebo-controlled 12-week study.

A total of 45 participants were screened to achieve the target enrollment of 28 eligible participants. A Consolidated Standards of Reporting Trials (CONSORT) flow diagram of participant enrollment, allocation, follow-up, and analysis is illustrated in Figure 2. The enrolled cohort had a mean age of 47.8 years and an average baseline BMI of 25.58 kg/m^2^. The randomized participant population consisted of participants who identified as White (71.4%), Asian (7.1%) and Black or African American (7.1%), and 14.3% of participants did not report their race. The female-to-male ratio was approximately 6:1 and was consistent between both study groups. Three participants voluntarily withdrew from the study.

At baseline, the average weight was 70.28 ± 10.186 kg, and the average diastolic and systolic blood pressure was 75.3 ± 6.12 and 112.1 ± 9.87 mmHg, respectively. The average heart rate was 66.3 ± 7.69 beats per minute, and the average respiratory rate was 14.1 ± 2.03 breaths per minute. There were no substantial differences in any vital signs or body measurements between the Muno-IgY and placebo groups at baseline. The baseline demographic and clinical characteristics of the participants in the Muno-IgY and placebo groups are given in Table 2.

Pregnancy tests for participants of childbearing potential conducted at Visits 2 and 6 were negative. Participants not of childbearing potential did not undergo testing.

Study product compliance was high, with a mean compliance rate of 98.79% across both treatment groups for the full study duration. No participant in either group demonstrated compliance below 80%.

For data analysis, the safety population included all participants who received at least one dose of the study product and was used for safety analysis. The full analysis set (FAS) population included all randomized participants who received at least one dose of the study product, had at least one baseline efficacy assessment, and one post-baseline efficacy assessment, and met all inclusion criteria. This population served as the primary data set for efficacy analysis. The per-protocol set (PPS), a subset of the FAS that included only participants who completed the study without major protocol deviations, was used to confirm the robustness of the FAS findings. The number of participants included or excluded in each analysis population is summarized in Table 3.

### 4.2. Effect of Muno-IgY on Anthropometrics, Vital Signs, and Clinical Laboratory Safety

No clinically meaningful changes in body weight, body mass index (BMI), or height were observed in either treatment group over the 12-week intervention period. Mean body weight and BMI remained stable from baseline to the end of the study in both the Muno-IgY and placebo groups, with no statistically significant between-group differences.

Vital signs, including systolic and diastolic blood pressure, heart rate, and respiratory rate, remained within normal physiological ranges throughout the study. No clinically relevant changes or treatment-related trends were observed at any assessment time point.

Hematology and clinical chemistry parameters remained stable across all visits. No participant demonstrated laboratory values outside clinically acceptable reference ranges that were considered related to the study product. There were no meaningful differences between treatment groups in hematological indices or clinical chemistry parameters, including liver and renal function markers.

### 4.3. Adverse Events and Safety Monitoring of Muno-IgY

A total of 16 participants experienced 28 treatment-emergent adverse events (TEAEs) during the study. In the Muno-IgY group, 7 participants (50.0%) reported 12 TEAEs, whereas in the placebo group, 9 participants (64.3%) reported 16 TEAEs. All TEAEs were mild in severity, and the majority (96.4%) were considered unrelated to the study products. Only one TEAE (ageusia) in the Muno-IgY group was suspected to be related to the study product. This event did not result in early termination; however, the participant withdrew three days after onset for reasons unrelated to the TEAE. The ageusia resolved after discontinuation, though the exact resolution time was not documented. Another participant reported dry mouth (unresolved), and one participant in the placebo group experienced a common cold with unknown outcome; both events were mild and deemed unrelated. All other TEAEs resolved by the end of the study.

The most frequently reported TEAE was flu-like symptoms, affecting four participants (14.3%, two in each group). Other commonly reported TEAEs included fatigue, upper respiratory infection, vasovagal reaction, headache, and loose stools. Three participants reported TEAEs consistent with upper respiratory tract infection (URTI) symptoms; however, none met the criteria for a clinical diagnosis of URTI by the QI. One Muno-IgY participant experienced transient rhinitis (1 h duration), and another had flu-like symptoms confounded by oral health issues, which were not classified as URTI.

No SAEs or deaths occurred during the study. A summary of TEAEs by study product group is presented in Table 4.

### 4.4. Effect of Muno-IgY on Immune Responses

#### 4.4.1. Incidence, Severity, and Duration of Upper Respiratory Tract Infections

In the FAS population, a total of 9 URTI episodes were reported across both study groups during the 12-week intervention. URTI symptoms were documented daily by participants in study diaries and subsequently evaluated by the Qualified Investigator (QI) to confirm a clinical diagnosis. Three participants experienced a treatment-emergent adverse event (TEAE) of URTI. All other episodes were identified by the QI based on symptom reports.

Overall, 14.3% of participants in the Muno-IgY group (*n* = 2) and 35.7% of participants in the placebo group (*n* = 5) experienced at least one URTI during the study period. Similar proportions were observed in the PPS population. Although the incidence of URTIs was numerically lower in the Muno-IgY group (approximately 59.9% reduction), the between-group difference was not statistically significant in the FAS or PPS population (*p* > 0.05). In the FAS population, the average incidence of URTI per participant over the 12-week study period was 0.1 ± 0.36 in the Muno-IgY group and 0.5 ± 0.85 in the placebo group, which was not statistically significant (*p* > 0.05). A summary of URTI occurrence by study group for the FAS population is presented in Table 5.

The average number of days of work or school missed due to URTI symptoms over 12 weeks was 0.0 days in the Muno-IgY group and 0.6 days in the placebo group. The average duration of reported URTIs over 12 weeks was 54.75 ± 41.37 h in the Muno-IgY group and 138.57 ± 205.43 h in the placebo group. As the incidence of URTIs across the study population was small, no statistical between-group comparison was made.

#### 4.4.2. Serum IgA Concentration

**Figure 3:**
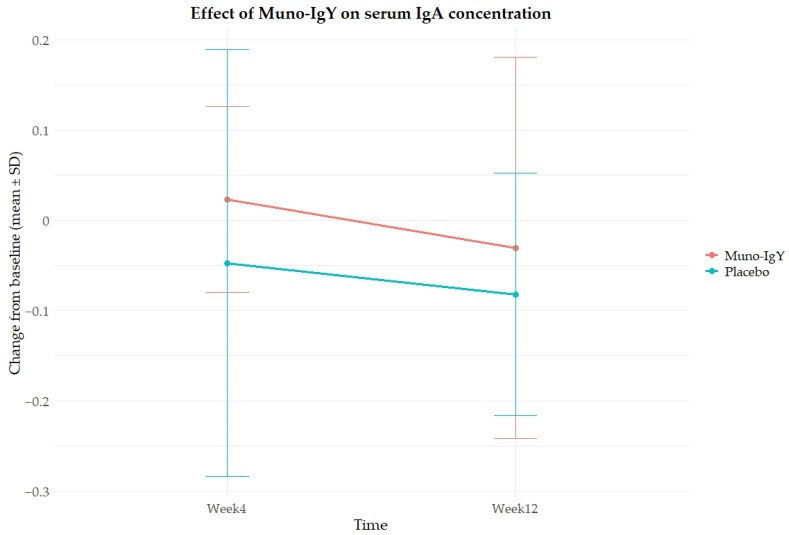
Effect of Muno-IgY on serum IgA concentration at week 4 and week 12 of the study. Data represent mean ± SD change from baseline. Repeated-measures ANOVA showed no significant effects of treatment, time, or interaction.

In the FAS population, an analysis of the effect of Muno-IgY on serum IgY concentration revealed that there was no main effect of treatment (*p* = 0.334), time (*p* = 0.253), or Treatment × Time interaction (*p* = 0.805). These results indicate that changes in serum IgA over 12 weeks were similar between Muno-IgY and Placebo and did not vary significantly between Week 4 and Week 12 (Figure 3).

#### 4.4.3. Inflammatory Biomarkers

**Figure 4:**
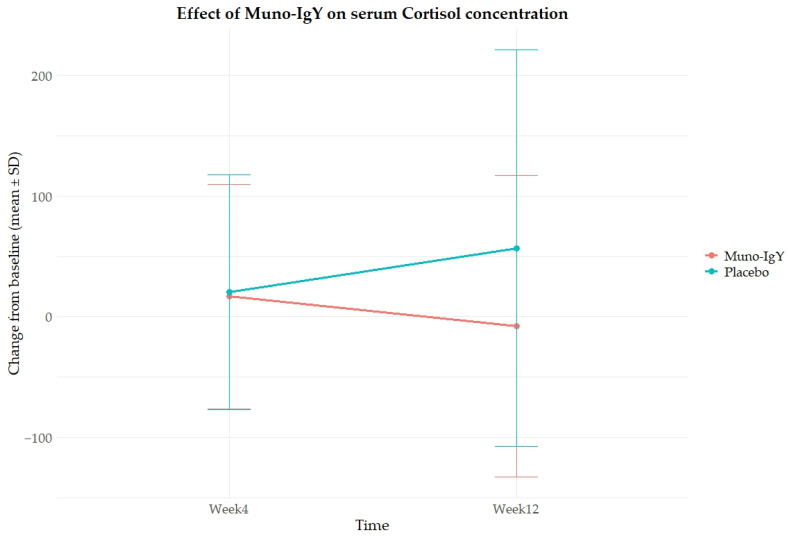
Effect of Muno-IgY on serum cortisol concentration at Week 4 and Week 12. Data are presented as mean change from baseline ± SD. Between-group comparisons were analyzed using a linear mixed-effects model with repeated measures.

In the FAS population, cortisol concentration, analyzed as a change from baseline, showed no statistically significant differences between the Muno-IgY and placebo groups at Week 4 (*p* = 0.945) or Week 12 (*p* = 0.201). Within-group comparisons showed no significant changes over time in either the Muno-IgY group (*p* = 0.483) or the placebo group (*p* = 0.284). Furthermore, the treatment-by-time interaction was not statistically significant (*p* = 0.215). Although a numerically greater reduction was observed in the Muno-IgY group at Week 12, due to high variability and small sample size, statistical significance was not achieved (Figure 4).

**Figure 5:**
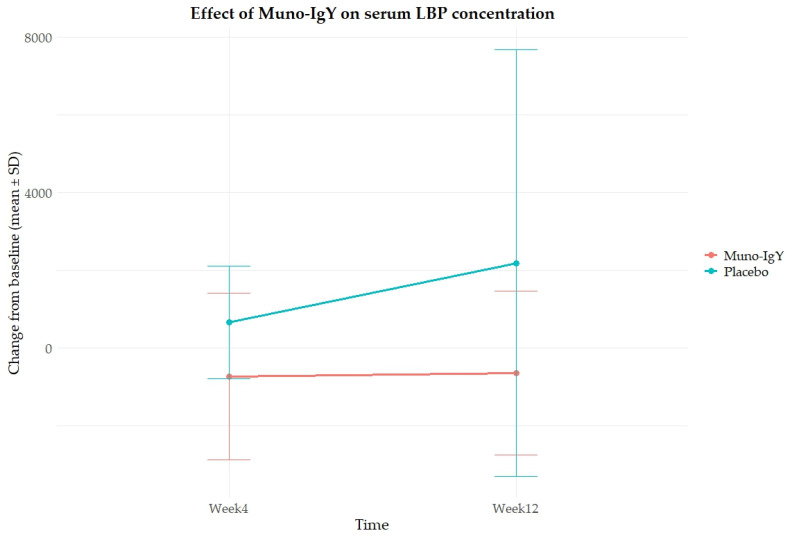
Effect of Muno-IgY on serum LBP concentration at Week 4 and Week 12. Data is presented as mean change from baseline ± SD. Between-group comparisons were analyzed using repeated-measures ANOVA with treatment as a between-subject factor and time as a within-subject factor.

The analysis of the effect of Muno-IgY on serum LBP concentration as a change from baseline over time (Week 4 and Week 12) revealed a significant main effect of treatment (*p* = 0.028) (Figure 5). Muno-IgY supplementation resulted in a significant overall reduction in serum LBP concentrations, expressed as change from baseline, compared with placebo. Mean LBP change from baseline was −729 ± 594 and −636 ± 607 at week 4 and week 12, respectively, in the Muno-IgY group, whereas placebo recipients exhibited increases of 674 ± 386 at week 4 and 2195 ± 1524 at week 12. This effect was independent of time (*p* = 0.504), with no significant treatment-by-time interaction (*p* = 0.446).

However, the effect of Muno-IgY supplementation on TNF-α over time revealed no significant treatment effect (*p* = 0.285), indicating that TNF-α levels did not differ between the Muno-IgY and placebo groups. Similarly, the time (*p* = 0.187) and treatment × time interaction (*p* = 0.187) effects were not significant, suggesting that TNF-α levels did not change significantly from Week 4 to Week 12 across all participants.

Similarly, for C-reactive protein (CRP), there was no significant effect of treatment (*p* = 0.504), time (*p* = 0.570), and treatment × time interaction (*p* = 0.154). IL-10 levels also showed no significant main effect of treatment (*p* = 0.983), time (*p* = 0.617), and treatment × time interaction (*p* = 0.598). No significant effect of treatment (*p* = 0.252), time (*p* = 0.522), or treatment × time interaction (*p* = 0.636) was observed for IL-1β concentration as well. IL-6 was not subjected to inferential statistical analysis due to a lack of within- and between-group variability across all study time points.

### 4.5. Effect of Muno-IgY on Post-Exercise Inflammatory, Immunity and Muscle Damage Response

#### 4.5.1. Serum IgA Concentration

Paired comparisons of serum IgA concentration before and 24 h after exercise showed a significant increase in the Muno-IgY group (mean change 0.125; *p* = 0.022), whereas the Placebo group showed a non-significant change (mean change 0.06; *p* = 0.197). This suggests that Muno-IgY supplementation may have attenuated exercise-induced decreases or supported maintenance of IgA levels following strenuous exercise.

**Figure 6:**
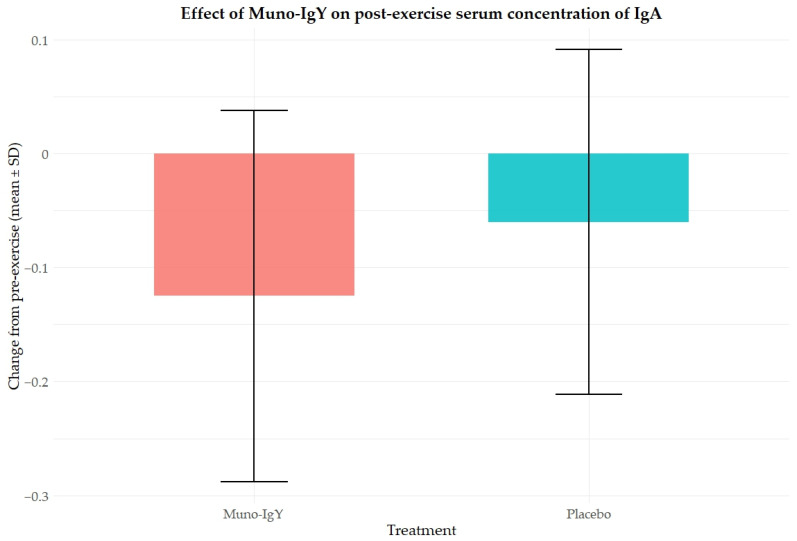
Effect of Muno-IgY on serum IgA concentration pre- and post-exercise. Data are presented as a change from pre-exercise baseline (mean ± SD). Statistical significance was assessed using a paired *t*-test; *p* < 0.05.

The pre- and post-exercise line graph (Figure 6) displays the mean change from pre-exercise with SEM for both groups, highlighting the significant rise in IgA for Muno-IgY and relatively stable levels in Placebo.

#### 4.5.2. Serum Markers of Muscle Damage

**Figure 7:**
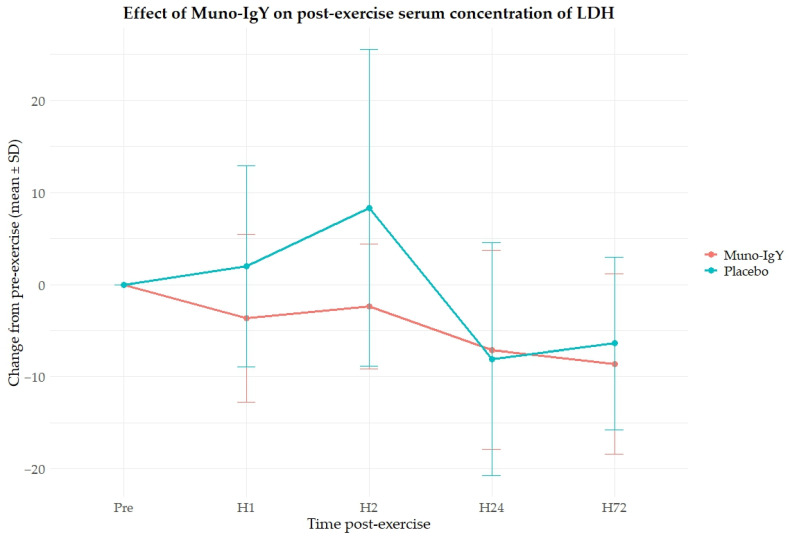
Changes in lactate dehydrogenase (LDH) levels from baseline (pre-exercise) at 1 h, 2 h, 24 h, and 72 h post-exercise in participants receiving Muno-IgY or placebo. Data are presented as mean ± SD.

In the FAS population, a significant main effect of group was observed on LDH concentration (*p* = 0.0365), indicating overall differences between the Muno-IgY and placebo groups (Figure 7). Also, a significant Time × Group interaction effect was observed (*p* = 0.0079), suggesting that LDH responses over time differed between treatments. However, there was no significant main effect of time on LDH concentration (*p* = 0.730). Post hoc pairwise comparisons revealed that at 1 h post-intervention, the Muno-IgY group exhibited a significantly greater reduction in LDH compared with placebo (*p* < 0.0001). This indicates an acute, time-specific effect of Muno-IgY on LDH levels.

For CK, no significant main effects of time (*p* = 0.246) or group (*p* = 0.513), and no significant interaction between time and group (*p* = 0.712) were observed. These results suggest that Muno-IgY supplementation did not have a significant influence on CK responses post-exercise.

#### 4.5.3. Serum Markers of Inflammation

The analysis of serum IL-1β concentration revealed no significant effects of time (*p* = 0.790), group (*p* = 0.689), or their interaction (*p* = 0.530). Overall, IL-1β levels remained stable across time and were not significantly affected by Muno-IgY supplementation.

There were also no significant differences between groups in the change from pre-exercise to any post-exercise time point for any other marker of inflammation, including CRP (*p* = 0.37), IL-10 (*p* = 0.48), cortisol (*p* = 0.19), LBP (*p* = 0.74) and TNF-α (*p* = 0.25). There was no measurable effect of exercise on IL-6 at any post-exercise time point, as values remained constant across all subjects and time points.

### 4.6. Effect of Muno-IgY Supplementation on Gut Microbiome

#### 4.6.1. Alpha Diversity

No significant differences in within-sample microbial diversity were observed between the Muno-IgY and placebo groups. Mean Shannon and Simpson diversity indices were comparable between groups (Table 6), indicating similar microbial richness and evenness across treatments.

#### 4.6.2. Beta Diversity

**Figure 8:**
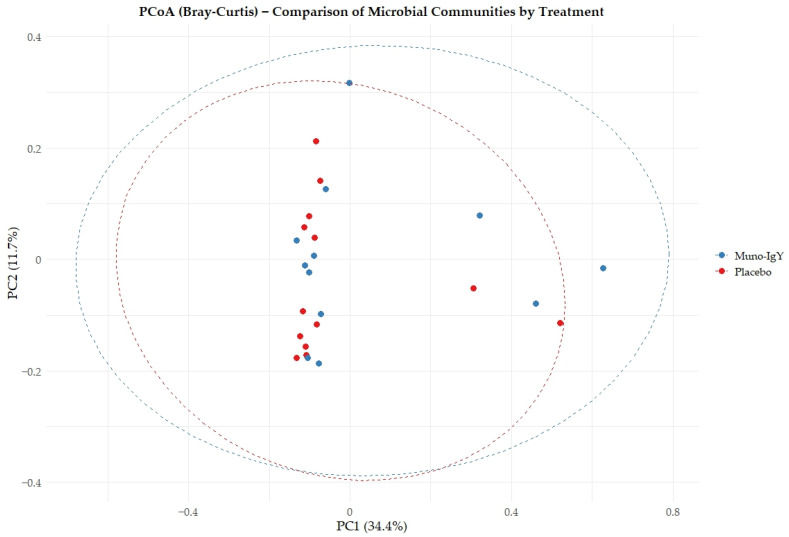
Principal coordinate analysis (PCoA) based on Bray–Curtis dissimilarity of fecal microbiome profiles from participants receiving Muno-IgY or placebo. Each point represents an individual participant. No significant separation between treatment groups was observed (PERMANOVA, *p* = 0.114).

Between-group differences in overall microbial community composition, assessed using Bray–Curtis’s dissimilarity, were not statistically significant. PERMANOVA analysis showed that treatment explained approximately 3.4% of the total variance in microbiome composition (R^2^ = 0.034), with no significant separation between groups (*p* = 0.114). Consistent with these findings, Principal coordinates analysis (PCoA) plots (Figure 8) demonstrated substantial overlap between the Muno-IgY and placebo groups, suggesting broadly similar global community structures.

#### 4.6.3. Species-Level Gut Microbiome Changes

**Figure 9:**
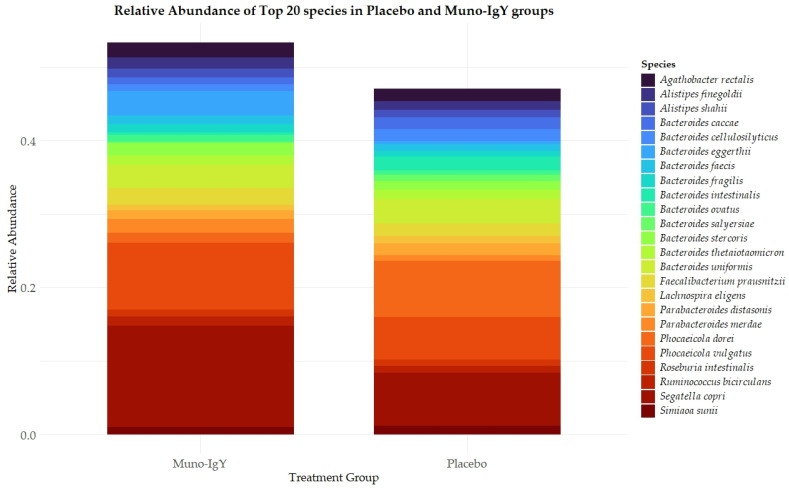
Top 20 most abundant bacterial species in fecal samples from participants receiving Muno-IgY or placebo. Mean relative abundances are shown for each species.

Relative abundances of the top 20 bacterial species in fecal samples were determined for participants receiving Muno-IgY or placebo (Figure 9). In the Muno-IgY group, *Segatella copri*, *Phocaeicola vulgatus*, and *Bacteroides eggerthii* were among the most abundant species, whereas in the placebo group, *Phocaeicola dorei*, *Segatella copri*, and *Phocaeicola vulgatus* predominated. Overall, the relative composition of the top species showed differences between treatment groups; however, no formal statistical comparisons were performed for individual species due to the exploratory nature of this study.

**Figure 10:**
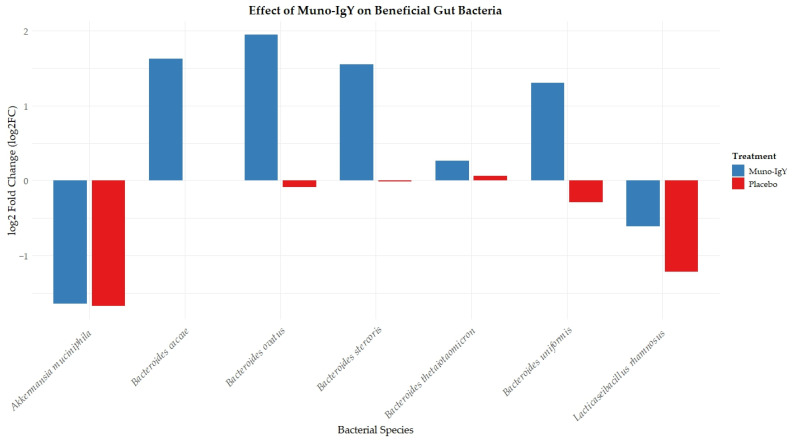
Change in relative abundance (log fold change) of selected beneficial gut bacterial species between Week 2 (baseline) and Week 12 (end of study) in participants receiving Muno-IgY or placebo.

An exploratory analysis of the gut microbiome was performed to assess changes in the relative abundance of selected bacterial species between baseline and the end of the study. Overall, no statistically significant differences were observed in alpha or beta diversity, and species-level analyses are presented for descriptive purposes. In exploratory analyses, Muno-IgY supplementation showed numerically higher relative abundance of several *Bacteroides* species (*B. caccae*, *B. ovatus*, *B. stercoris*, *B. uniformis*, *B. thetaiotaomicron*) compared with placebo. *Akkermansia muciniphila* exhibited similar reductions across groups, while *Lacticaseibacillus rhamnosus* appeared to decrease to a lesser extent in the Muno-IgY group (Figure 10).

**Figure 11:**
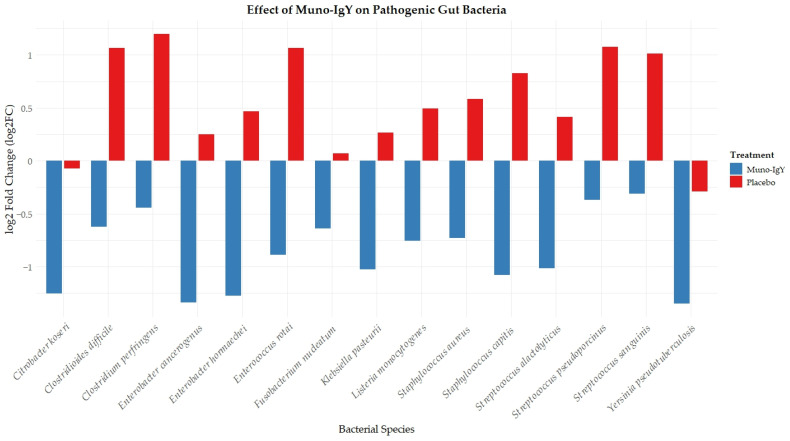
Change in relative abundance (log fold change) of selected pathogenic and opportunistic gut bacterial species between Week 2 (baseline) and Week 12 (end of study) in participants receiving Muno-IgY or placebo.

Conversely, several bacterial taxa that are commonly classified in the literature as opportunistic or host-associated species, including *Clostridioides difficile*, *Clostridium perfringens*, *Fusobacterium nucleatum*, *Enterobacter* spp., *Citrobacter koseri*, *Klebsiella* spp., *Streptococcus* spp., *Enterococcus rotai*, *Yersinia* spp., *Staphylococcus* spp., and *Listeria monocytogenes*, demonstrated numerically lower relative abundance in the Muno-IgY group relative to placebo (Figure 11). Similar directional trends were observed across other gut-associated taxa frequently described in the literature in relation to short-chain fatty acid production or lactic acid metabolism, including *Faecalibacterium prausnitzii*, *Roseburia* spp., *Anaerostipes hadrus*, *Coprococcus* spp. and members of the genera *Lactococcus* and *Leuconostoc*. All findings are exploratory and descriptive, as no statistically significant differences were detected between the groups.

**Figure 12:**
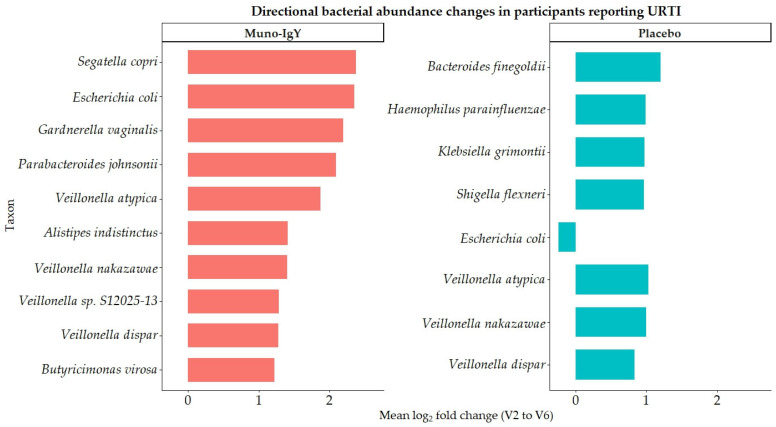
Directional changes in bacterial abundance in infected participants receiving Muno-IgY or placebo. Horizontal bars represent mean log2 fold-change (V2 to V6) per taxon.

Also, to assess the effect of treatment on the gut microbiome in participants with at least one incidence of URTI during the study period, baseline and post-intervention samples were analyzed using within-subject fold-change and log2 fold-change calculations, followed by group-level summarization by infection status and treatment. Analyses were restricted to infected participants and taxa exhibiting a mean fold change ≥ 3. The summarized data are presented in Table 7, and the directional shifts in bacterial abundance are visualized in Figure 12.

Only two participants in the Muno-IgY group and five participants in the placebo group reported at least one incidence of URTI during the study period; the small number of affected participants in the Muno-IgY group may limit the generalizability and interpretation of the observed microbiome changes. Hence, microbiome analyses were exploratory and purely descriptive. In the placebo group, several bacterial taxa exhibited large mean fold increases, including *Escherichia coli*, *Klebsiella grimontii*, and *Shigella flexneri*; however, these changes were accompanied by pronounced discrepancies between mean and median values, with median log2 fold changes near or below zero. This pattern indicates substantial inter-individual variability and suggests episodic or outlier-driven microbial blooms rather than uniform shifts across subjects.

In contrast, exploratory analyses suggested directional enrichment of multiple bacterial taxa in the Muno-IgY group, as reflected in similar mean and median fold-change and log2 fold-change values. Notably, taxa linked to anaerobic metabolism and gut ecosystem structure, including *Segatella copri*, *Parabacteroides johnsonii*, *Alistipes indistinctus*, and *Butyricimonas virosa*, demonstrated coordinated increases. Members of the *Veillonella* genus showed uniform enrichment exclusively in the Muno-IgY group. While similar mean and median fold-change values were noted for these taxa within the Muno-IgY group, these observations are highly vulnerable to outlier effects and inter-individual variability. Consequently, these findings do not support conclusions regarding microbiome stabilization or treatment-related enrichment, but rather serve to inform hypotheses for future, adequately powered studies.

## 5. Discussion

This randomized, double-blind, placebo-controlled pilot study provides an exploratory assessment of the effects of Muno-IgY supplementation on respiratory health, immune function, exercise-induced physiological stress, and gut microbiome composition in healthy adults. While many outcomes did not reach statistical significance, exploratory analyses across clinical, biochemical, and microbiome endpoints revealed numerical patterns that may be biologically relevant. However, given the pilot nature of the study, small sample size, and limited event counts, these observations should not be interpreted as evidence of consistent or treatment-driven effects. Instead, they highlight areas for targeted investigation in future trials specifically designed to evaluate these outcomes with sufficient statistical power.

In this study, participants receiving Muno-IgY reported a numerically lower incidence of URTIs, shorter symptom duration, and fewer missed work or school days compared with placebo, though differences were not statistically significant. Although a numerically lower URTI incidence was observed, the small number of events and lack of statistical significance limited meaningful comparison with prior studies demonstrating the protective effect of IgY, particularly in populations exposed to environmental or physiological stressors in animal models [24,25,26]. The low overall number of URTI events, combined with the modest sample size, limited the statistical power to detect between-group differences. As this was an exploratory pilot study, the primary objective was to assess feasibility, safety, and signal directionality rather than definitive efficacy. While mechanistic hypotheses exist, the present data do not allow confirmation of biological effects [15]. Unlike pathogen-specific IgY formulations studied previously, Muno-IgY is hypothesized to have mucosal activity, which warrants further investigation.

Though there was a numerical reduction in the URTI incidence in subjects supplemented with Muno-IgY, no significant changes were observed in resting serum IgA concentrations or serum concentrations of inflammatory cytokines over the 12-week intervention period. This is consistent with previous nutritional intervention studies in healthy adults, where baseline immune markers often remain stable due to intact immune homeostasis [27]. In such populations, supplements may exert their effects primarily under conditions of immune challenge rather than at rest.

Interestingly, Muno-IgY supplementation resulted in a significant reduction in serum LBP levels, an acute-phase reactant associated with gut-derived endotoxemia and systemic immune activation. Elevated LBP levels have been linked to increased intestinal permeability, chronic low-grade inflammation and inhibit the immuno-stimulatory effects of bacterial components [28]. However, changes in LBP alone should be interpreted cautiously and cannot be used to infer gut barrier function in this pilot study.

Exercise is a well-established physiological stressor that can transiently suppress immune function and increase susceptibility to infections, particularly through reductions in mucosal IgA [29]. In the present study, Muno-IgY supplementation led to a significant increase in serum IgA 24 h post-exercise, whereas no significant change was observed in the placebo group. To our knowledge, there are no published human studies that have specifically examined the effect of oral IgY supplementation on serum IgA concentrations in athletes or healthy adults post-exercise, and exercise-related applications of IgY have thus far remained largely theoretical or confined to unrelated immune contexts. Nevertheless, the broader exercise immunology literature demonstrates that both acute and chronic physical exercise can independently modulate immunoglobulin levels, including IgA, particularly in physically active or high-training-load populations [29]. In this context, comparable patterns have been reported with other gut-targeted nutritional interventions. For example, a recent clinical study investigating probiotic supplementation in physically active individuals observed increases in serum IgA following exercise, despite minimal changes in systemic inflammatory markers [30]. Although probiotics and IgY act through distinct biological mechanisms, both interventions are thought to primarily influence gut microbiota and associated mucosal immune function, supporting immune resilience during periods of physiological stress. These observations provide a relevant framework for interpreting the immune biomarker responses observed in the present IgY intervention study.

Lactate dehydrogenase is a cytosolic enzyme that leaks into the circulation following mechanical and metabolic stress to muscle tissue. Its serum activity increases significantly in the setting of non-traumatic acute muscle injury and rhabdomyolysis, reflecting cellular membrane disruption and tissue stress rather than direct pathology [31]. In the present study, Muno-IgY supplementation was associated with a favorable acute reduction in LDH levels 1 h post-exercise compared with the placebo, which may indicate a preliminary effect on exercise-induced muscle stress or an enhancement of early recovery processes. While CK did not differ between groups, LDH has been reported to respond more rapidly to physiological stress and may provide complementary information about acute tissue perturbation following exertion [32]. Together, these findings suggest that Muno-IgY may buffer aspects of exercise-induced physiological strain, potentially through immune modulation or reduced inflammatory signaling that limits cellular leakage of LDH into the circulation.

The gut microbiome diversity metrics did not differ significantly between groups, which is consistent with prior findings in human intervention studies showing that short-term dietary or exercise interventions often do not yield large, statistically significant changes in overall gut microbial diversity, particularly in modestly sized cohorts [33]. However, exploratory analyses showed descriptive differences in microbial composition associated with Muno-IgY supplementation. These included numerically higher relative abundances of commensal taxa such as *Bacteroides* species and reduced abundance of several opportunistic or potentially pathogenic taxa. Although studies of orally administered IgY and human microbiota remain limited, evidence from gut immunology demonstrates that mucosal immunoglobulins can interact directly with microbial communities, influencing the relative abundance of specific taxa without broadly disrupting beneficial commensal populations [33]. Such selective modulation is biologically plausible given IgA’s role in stabilizing host-microbiota symbiosis by binding microbial antigens and promoting niche adherence of commensals, with analogous effects potentially attributable to exogenous IgY at the mucosal surface [34]. The reduction in opportunistic taxa alongside preservation of beneficial microbes may support gut barrier integrity and facilitate immune regulation, aligning with models of gut–lung immune crosstalk wherein gut microbial composition influences systemic and distal mucosal immunity [35]. As this study was not powered for formal microbiome inference, these findings should be interpreted as hypothesis-generating and warrant further investigation in larger, mechanistically targeted trials.

Although the small number of participants reporting URTI, particularly in the Muno-IgY group, limits generalizability, the observed patterns suggest targeted modulation of the gut microbiome. Episodic blooms of opportunistic taxa in placebo participants are consistent with prior reports of transient Enterobacteriaceae expansions during immune stress [36]. In contrast, Muno-IgY supplementation appeared to help maintain key anaerobic and commensal taxa, supporting the overall microbial ecosystem. These findings align with evidence that orally delivered immunoglobulins can selectively influence microbial composition without broadly disrupting diversity, analogous to IgA-mediated mucosal regulation [37]. Overall, these findings are descriptive and hypothesis-generating. Larger, adequately powered studies are required to confirm the role of Muno-IgY in stabilizing the gut microbiome during periods of immune stress.

As hypothesized, in this pilot study, Muno-IgY was well-tolerated, and the safety profile was favorable. A total of 16 participants reported 28 TEAEs, the majority of which were mild and deemed unrelated to the study products. Only one TEAE (ageusia) in the Muno-IgY group was suspected to be related to the study product, and it resolved after discontinuation. No SAEs or deaths occurred, and no participants discontinued due to adverse events. The most frequently reported TEAEs were flu-like symptoms, fatigue, upper respiratory infection, vasovagal reaction, headache, and loose stools, with similar distribution across the Muno-IgY and placebo groups. These findings demonstrate that Muno-IgY is safe and well-tolerated in healthy adults over 12 weeks.

Key strengths of this study include its comprehensive, systems-level approach integrating clinical outcomes, immune and inflammatory biomarkers, exercise physiology, and shotgun metagenomic analysis. The randomized, double-blind design and high compliance further strengthen the validity of the findings. Limitations of this pilot study include the small sample size and the absence of a formal a priori power calculation. Consequently, all analyses should be considered exploratory, and the results are susceptible to both Type I and Type II errors. Additionally, the high predominance of female participants and the resulting small subgroup sizes may limit the generalizability of the findings to broader populations.

Findings, particularly for URTI incidence and microbiome outcomes, should be interpreted with caution and confirmed in larger, adequately powered studies. In addition, eligibility was based on participants’ self-reported history of at least one URTI in the previous six months. This may introduce misclassification or selection bias, potentially affecting internal validity. However, all URTI episodes occurring during the study were prospectively documented in daily diaries and subsequently evaluated by the Qualified Investigator to confirm clinical relevance. While self-report is commonly used in studies involving generally healthy adults [38,39], the small number of URTI events suggests that numerical differences between groups should be interpreted cautiously. The microbiome analyses were exploratory and descriptive.

Despite these limitations, directional trends across multiple domains suggest that Muno-IgY may support immune resilience, respiratory health, and recovery from physiological stress. These findings warrant larger, adequately powered trials in populations at higher risk of URTIs, such as athletes, older adults, or individuals under chronic stress. Future studies should explore longer intervention durations, pathogen-specific outcomes, and mechanistic links between IgY supplementation, gut barrier function, and immune regulation.

## 6. Conclusions

In this exploratory pilot study, Muno-IgY supplementation was safe and well-tolerated. Numerical trends in respiratory outcomes, immune markers, exercise-induced physiological stress, and gut microbial composition were observed, though most did not reach statistical significance. These findings provide preliminary signals that can inform future, adequately powered studies to further investigate the potential effects of Muno-IgY in populations at higher risk of URTIs or physiological stress.

## Figures and Tables

**Table 1:** Data elements collected for each adverse event to support consistent documentation, safety evaluation, and regulatory reporting.

Summary of Adverse Event Data Elements Collected During the Study	
Parameter	Description
Event description	Brief description of symptoms or diagnosis
Onset/End	Date and time of event start and resolution
Severity	Mild: no limitation; Moderate: some limitation; Severe: unable to perform usual activities
Action taken	Dose unchanged, reduced, interrupted, withdrawn, or other actions
Outcome	Fatal, recovered/resolved, recovered with sequelae, not resolved, unknown
Causality	Related, suspected, or not related to the study product
Pregnancy monitoring	Abnormal pregnancy outcomes were considered AEs/SAEs. Participants with pregnancy were withdrawn or followed to resolution.

**Table 2:** Baseline demographic and clinical characteristics of participants in the randomized controlled trial.

Characteristic	Muno-IgY (*n* = 14)	Placebo (*n* = 14)	Total (*N* = 28)
Age, years			
Mean ± SD	47.5 ± 7.1	48.1 ± 8.2	47.8 ± 7.6
Range	36–58	35–65	35–65
Sex, *n* (%)			
Female	12 (85.7)	12 (85.7)	24 (85.7)
Male	2 (14.3)	2 (14.3)	4 (14.3)
Race, *n* (%)			
White	10 (71.4)	10 (71.4)	20 (71.4)
Asian	1 (7.1)	1 (7.1)	2 (7.1)
Black or African American	1 (7.1)	1 (7.1)	2 (7.1)
Not reported	2 (14.3)	2 (14.3)	4 (14.3)
Ethnicity, *n* (%)			
Not Hispanic or Latino	12 (85.7)	12 (85.7)	24 (85.7)
Hispanic or Latino	2 (14.3)	2 (14.3)	4 (14.3)
BMI (kg/m^2^)			
Mean ± SD	25.4 ± 2.7	25.8 ± 2.3	25.6 ± 2.5
Range	20.2–29.5	21.3–29.8	20.2–29.8
Vital signs			
Systolic BP (mmHg), mean ± SD	110.9 ± 10.4	113.3 ± 9.5	112.1 ± 9.9
Diastolic BP (mmHg), mean ± SD	74.9 ± 5.8	75.6 ± 6.6	75.3 ± 6.1
Heart rate (bpm), mean ± SD	65.4 ± 7.9	67.2 ± 7.6	66.3 ± 7.7
Respiratory rate (breaths/min), mean ± SD	14.4 ± 2.2	13.9 ± 1.9	14.1 ± 2.0

**Table 3:** Disposition of Participants and Analysis Sets.

	Placebo	Muno-IgY	Total
Screened			45
Met criteria but not randomized			2
Screening failure			15
Randomized	14 (100%)	14 (100%)	28 (100%)
Included in the Safety population	14 (100%)	14 (100%)	28 (100%)
Included in the FAS population	14 (100%)	14 (100%)	28 (100%)
Included in the PPS population	12 (85.7%)	13 (92.9%)	25 (89.3%)
Completed study	12 (85.7%)	13 (92.9%)	25 (89.3%)
Excluded from PPS	2 (14.3%)	1 (7.1%)	3 (10.7%)
Withdrawal by subject	2 (14.3%)	1 (7.1%)	3 (10.7%)

**Table 4:** Summary of treatment-emergent adverse events in healthy adults during 12-week supplementation with Muno-IgY or placebo (Safety population, *N* = 28).

		Muno-IgY (*n* = 14)	Placebo (*n* = 14)	Total (*N* = 28)
Overall		7 (50%)	9 (64.3%)	16 (57.1%)
Relation	Related	0 (0%)	0 (0%)	0 (0%)
	Suspected	1 (7.1%)	0 (0%)	1 (3.6%)
	Not related	6 (42.9%)	9 (64.3%)	15 (53.6%)
Severity	Severe	0 (0%)	0 (0%)	0 (0%)
	Moderate	0 (0%)	0 (0%)	0 (0%)
	Mild	7 (50%)	9 (64.3%)	16 (57.1%)
Discontinuation	0 (0%)	0 (0%)	0 (0%)	0 (0%)

**Table 5:** URTI occurrence by study product over 12 weeks in the FAS population.

Number of Participants			Percentage of Participants with URTI			
Muno-IgY	Placebo	Total	Muno-IgY	Placebo	Total	*p*-Value
2	5	7	14.3%	35.7%	25.0%	0.38

**Table 6:** Alpha diversity indices of fecal microbiota in the Muno-IgY and placebo groups. Values are presented as mean ± SD.

Diversity Index	Placebo	Muno-IgY	*p*-Value
Shannon	3.38 ± 0.57	3.43 ± 0.75	0.59
Simpson	0.86 ± 0.08	0.85 ± 0.12	0.53

**Table 7:** Mean and median fold-change and log2 fold-change in bacterial taxa in infected participants (fold change ≥ 3). Values represent changes from baseline (Week 2) to post-intervention (Week 6) for participants receiving either Muno-IgY or placebo. Taxa are ordered by mean log2 fold change.

Taxon	Treatment	Mean Fold Change	Median Fold Change	Mean log2FC	Median log2FC
*Escherichia coli*	Placebo	14.36	0.33	−0.24	−1.62
*Klebsiella grimontii*	Placebo	12.87	0.92	0.97	−0.12
*Shigella flexneri*	Placebo	12.27	0.96	0.96	−0.06
*Bacteroides finegoldii*	Placebo	3.59	1.99	1.20	0.99
*Haemophilus parainfluenzae*	Placebo	4.01	2.35	0.99	1.23
*Veillonella atypica*	Placebo	3.99	1.60	1.02	0.68
*Veillonella dispar*	Placebo	3.89	1.19	0.83	0.25
*Veillonella nakazawae*	Placebo	6.62	1.30	1.00	0.38
*Segatella copri*	Muno-IgY	11.47	11.47	2.37	2.37
*Parabacteroides johnsonii*	Muno-IgY	6.57	6.57	2.09	2.09
*Escherichia coli*	Muno-IgY	5.92	5.92	2.35	2.35
*Gardnerella vaginalis*	Muno-IgY	8.49	8.49	2.19	2.19
*Veillonella atypica*	Muno-IgY	4.39	4.39	1.87	1.87
*Veillonella dispar*	Muno-IgY	3.28	3.28	1.28	1.28
*Veillonella nakazawae*	Muno-IgY	4.38	4.38	1.40	1.40
*Veillonella* sp. *S12025-13*	Muno-IgY	4.46	4.46	1.28	1.28
*Butyricimonas virosa*	Muno-IgY	3.20	3.20	1.22	1.22
*Alistipes indistinctus*	Muno-IgY	4.87	4.87	1.41	1.41

## Data Availability

The original contributions presented in this study are included in the article. The raw data supporting the conclusions of this manuscript will be made available by the authors upon reasonable request. Further inquiries can be directed to the corresponding author.
